# Supplementary material for: Balance rehabilitation with a virtual reality protocol for patients with hereditary spastic paraplegia: Protocol for a clinical trial
Source: PLoS One. 2021 Apr 1;16(4):e0249095. doi: 10.1371/journal.pone.0249095 (PMC8016341; doi:10.1371/journal.pone.0249095)
Supplement: S1 File — (DOCX) [file pone.0249095.s003.docx]

**Balance rehabilitation with a virtual reality protocol for patients with hereditary spastic paraplegia: Protocol for a clinical trial**

**Design**

A randomized, controlled, clinical trial will be conducted in accordance with the ethical principles governing research involving human subjects stipulated in Resolution 466/2012 of the Brazilian National Board of Health. The study received approval from the Human Research Ethics Committee of the *Faculdade Evangélica Mackenzie do Paraná* (process number: 37083714.0.0000.0103; certificate number: 3.580.973) and the protocol was registered and approved on the Rebec platform, trials RBR-3JMX67. All patients agreed to participate by signing a statement of informed consent.

A person participant of the team, not directly involved in the research, will be responsiblefor obtainingthe signatures oftheconsentforms of patients which were initially selected as potential participants in the trials.

**Study population**

Forty patients with a diagnosis of HSP will be recruited from the hospital of the Federal University of Parana in the city of Curitiba, Brazil. To participate, the volunteers must meet the following eligibility criteria:

**Inclusion criteria**

Age 18 years or older (with no restriction imposed regarding maximum age);

Men or women with HSP.

**Exclusion criteria**

Otologic condition that could affect the vestibular examination;

Use of gait-assistance device;

Inability to understand simple verbal commands;

Significant musculoskeletal condition that could impede the assessment and VR;

Severe visual impairment or other abnormality that could impede the proposed procedures.

Eligible patients will undergo the following:

**Patient history**

Patient histories will be taken with an emphasis on otoneurological signs and symptoms;

**Otolaryngologic evaluation**

The aim of the otolaryngologic assessment is to exclude individuals with any abnormality that could interfere with the study;

**Vestibular assessment**

Subsequently, they will carry out the evaluation by the physiological profile (PPA) and Lafayette dynamometer, and application of 10 questionnaires / scales described below before the rehabilitation (1st evaluation) and after the 20th rehabilitation session 2nd assessment), to observe the post- intervention.

**Physiological Profile Assessment**

The physiological profile assessment (PPA) is a validated assessment tool for the risk of falls developed by Lord, Menz and Tiedemann *et al*.^12^. Performs direct assessment of sensory motor skills. The score indicates the degree of risk: <0 = low risk, 0-1 = mild risk; 1-2 = moderate risk; and> 2 = high risk of falls, indicating the individual's performance in comparison to the normative values for each age group.

**Lafayette dynamometer**

The Lafayette dynamometer^13,14^ is a portable device for the objective quantification of muscle strength based on the assessment of maximum voluntary isometric contraction^15^. Applicable to all segments of the body, measures peak force, duration of peak force, force (kgf) between selected intervals, mean force, total test time and peak torque.

**Quality of life**

The Quality of Life Group of the World Health Organization (WHO) developed the short version of the WHOQOL-100 denominated the WHOQOL-Bref ^16^. This version is a useful alternative to the long version, as the proposed study will involve several assessment tools. The questionnaire is used to determine the perceptions of individuals about your health and can be used to assess the quality of life of different populations and in different situations^17.^

**Berg Balance Scale (BBS)**

The Brazilian version of the BBS, which was cross-culturally adapted to the Brazilian population by Myamoto *et al.*^18^, will be used to determine risk factors for the loss of independence and falls. The maximum score is 56 points, with higher score denoting better balance. The score is analyzed to determine the degree of the risk of falls: low, medium, high or 100% risk.

**ABC Activities-Specific Balance Confidence (ABC) scale**

The ABC scale, translated and adapted to the Brazilian population^19^, showing good quality, discriminating validity, consistency and reliability^20^.It has 16 items for assessing balance during a set of activities of daily living (ADLs) of medium difficulty^21^. The confidence for each ADL is measured by the volunteer, choosing a percentage point on a scale ranging from 0% (without confidence) to 100% (total confidence). A total greater than 80% corresponds to a high functional level, 50 to 80% moderate physical functioning and a score below 50% indicates a low functional level^22^.

**Vestibular Disorders Activities of Daily Living (VADL) scale**

The VADL scale was developed by Cohen and Kimball^23^ to assess the impact of dizziness and imbalance on the performance of activities of daily living among individuals with vestibular disorders. The scale involves 28 activities divided into three dimensions: functional, locomotion and instrumental. The total VADL and dimension scores are determined by the median of the activity scores, with higher scores denoting greater dependence and disability^24^. The translation of the VADL from English into Brazilian Portuguese was performed following the guidelines of the Process of Cross-Cultural Adaptation^25^.The Brazilian version had adequate reliability and is a new assessment tool in the country for investigating functional capacity in individuals with vestibulopathies as well as guiding therapeutic planning, particularly vestibular rehabilitation^26^.

**Mini Mental State Examination (MMSE)**

The MMSE is a fast, short, 30-item measure to screen for cognitive impairment^27^ that addresses spatiotemporal orientation, immediate recall and word evocation, calculation, naming, repetition, executing a command, reading, writing and visuomotor skill^28-30^.The MMSE items were categorized by Folstein *et al*.^27^into five dimensions based on theoretical analysis and clinical practice. The Brazilian version of the MMSE was adapted by Brucki *et al*.^29^, who described the adaptations for the use of MMSE in Brazil and proposed rules to standardize its application for the detection of cognitive loss in the follow up of disease and monitoring of the response to treatments. The measure received the denomination “mini” because it focuses on only the cognitive aspects of mental function, excluding mood and abnormal mental functions^27^.It provides information on different cognitive variables^31^, containing items grouped into seven categories, each of which is designed to evaluate specific cognitive functions. The total ranges from 0 points (severe cognitive impairment) to 30 points (best cognitive performance).

**Timed Up and Go (TUG) test**

According to Paula, Alves Jr. and Prata^32^, the TUG test consists of standing up from a chair without using the arms, walking three meters, turning around, walking back to the chair and sitting down again. The test begins and ends with the volunteer seated with the back against the backrest of the chair. The volunteer begins with the command "go" and the time needed to complete the task is timed with a stopwatch. According to the literature, there is no consensus on the results of this test. However, Guimarães *et al*.^33^ established the following markers: less than 10 seconds to complete the TUG test indicates a low risk of falls; 10 to 20 seconds indicates a medium risk and more than 20 seconds indicates a high risk of falls.

**Falls Efficacy Scale – International (FES-I)**

The FES-I is a self-administered questionnaire designed to evaluate the fear of falling during a set of activities. It is composed of 16 items scored from 1 to 4 points, with higher scores denoting a great fear of falling during a given activity. The FES-I has internal consistency of 0.96. Moreover, most items enable differentiating individuals who have suffered a fall, those who have suffered two or more falls and those who have not fallen^34^.The version validated for the Portuguese language will be used^35^.

**Visual Analog Scale (VAS)**

The VAS is a one-dimensional scale used to measure pain intensity consisting of a 10-cm horizontal line with zero at one end (accompanied by the expression "no pain") and 10 at the other end ("unbearable pain"). Pain will be classified as mild (1 to 3 cm), moderate (4 to 6 cm) or strong (7 to 9 cm). The absence of pain (score: 0) indicates that the volunteer has no difficulties performing activities of daily living; mild pain indicates the ability to perform activities despite pain; moderate pain partially or completely hinders activities and performance is compromised; and strong pain completely impedes the execution of activities^36^.

**Randomization**

Prior to the intervention, the volunteers will be randomly allocated to two different groups. Randomization will be performed by an independent researcher using a simple lottery system with sealed opaque envelopes immediately after the baseline assessment. The volunteers will be considered participants in the study the moment the envelope is opened. The participants will be randomized to receive the following distinct interventions:

Group I: Twenty volunteers will undergo VR with virtual reality (balance games) using the Wii® console, Wii-Remote and Wii Balance Board (Nintendo).

Group II: Twenty volunteers will undergo VR with virtual reality (balance games and muscle strength games) using the Wii® console, Wii-Remote and Wii Balance Board (Nintendo).

The games will last from 30 minutes to one hour, with sessions twice a week for 10 weeks (total: 20 sessions).

**Treatment**

All participants will receive orientation regarding the rehabilitation procedure. The VR will involve virtual reality with the use of the Wii Fit Plus®, Wii-Remote and Wii Balance Board (Nintendo). The Wii Balance Board (WBB) is a platform with sensors that detect the position and orientation of the gamer. In some games, the player must perform the same movement he/she would do in a real game. The VR with virtual reality games will be performed in both groups for 30 minutes twice a week for a total of 20 sessions. Group I will play five balance games. Group II will play the same five balance games plus four strength games. The games will be selected to favor changes in balance and postural instability. After completing the collection of all data, to ensure the same rehabilitation for both groups, will be available the training with strength exercises to group I.

The following are the balance games performed on the WBB:

Soccer Heading®

Table Tilt®

Tightrope Walk®

Penguin Slide®

Perfect 10®

The muscle strengthening exercises were selected to improve balance, which depends on the interaction between vision, vestibular and peripheral sensations, central commands and neuromuscular responses:

Single leg extension®

Torso Twist®

Sideways Leg Lift®

Single Leg Twist®

The training phases will be performed simultaneously, avoiding complications and alterations that may compromise the validity of training. The assessment will be performed again after the 20 intervention sessions.

The time schedule of enrolment, interventions, assessments and visits for participants are describe in Table 2 in accordance of Spirit checklist (Figure 1)

**Management**

The researches ensure that the anonymous character and the confidentialityof participants, dealing with people or data files, will be maintained and their identities or any kind of identification will be protected. The clinics records, research instruments or any document that can be used, containing data of participants, will not be identified by the name, but through a code, even when submitted to regulatory institutions or sponsors. Researchers willmaintain the record of inclusion of participants confidentially, containing codes, names and addresses for their own use. The files of the Consent forms will be issued in two copies each one of them to be known and signed by participants, researchers and researched (or legal representatives), the files will be maintained by the researcher in strict confidence, all together, in a single file related to that research. We ensure that participants that comprise the casuistry will receive an original copy of the Consent term.

There is no personal expense for the participant in any stage of the study, instead, all assessment shall be free, including the otorhinolaryngological evaluation, PPA and dynamometer. There is also no financial compensation related to your participation. If there is any additional expense, it will be absorbed by the research budget. Participation in this study is completelyvoluntary and non-participation does not imply any change in your medical follow-up, not even will change the relationship between the team and the patient. After signing the Consent term, you will have total liberty to take it out at any moment and leave participatingin the study, if desired, without any damage to the development of the treatment and follow-up at the institution.

The results of this research may be presented in at meetings or publications, however, your identity will not be revealed in these presentations.

The risks during the research are minimal or non-existent, but the realization of the assessment and rehabilitations may cause discomfort, arising from the direct interference on the vestibular system responsible for the balance. The support in this sense may be momentary interruption of the evaluation VR, until medical interventions if necessary. However, if persists the discomforts the participant will be removed from the research and the data collected will be excluded from the sampling, and the patient will not be reallocated in any group.

As a strategy to improve the adhesion to intervention protocols, will be promoted phones contacts for confirmations of treatments sessions, as well as will be available through reports the results of assessment which the participants are submitted.

The only intervention not permitted will be related to the conventional vestibular rehabilitation and virtual.

This work was supported by the authors by themselves.

**Quality assurance**

The treatment protocol will be performed by two skilled researchers in the field of physical therapy/physical education who have previously performed this protocol with patients with neurodegenerative diseases (Spinocerebellar ataxia and Parkinson's disease) and will not be aware of the results of the initial evaluations of the patients who will undergo the interventions.

**Assessment of results**

The participants will be evaluated by an independent examiner who will be blinded to the allocation to the different groups. The primary outcomes will be evaluated with each of the assessment tools, considering an improvement in the performance in the comparison of the pre-intervention and post-intervention assessment:

PPA: quantifies the risk of falls.

Dynamometer: verification of improvement in muscle strength;

WHOQOL-Bref: improvement in perceived quality of life in different domains;

BBS: analysis of balance status;

ABC: like BBS, verifies balance, but related to activities of daily living;

VADL: investigates impact of dizziness and balance on degree of dependence;

MMSE: measures degree of cognitive impairment;

TUG test: measures risk of falls related to muscle weakness;

FES-I: investigates fear of falling;

VAS: quantifies pain intensity.

The secondary outcomes will quantify the improvement in performance on the assessment. For such, a specific questionnaire will be administered for each assessment.

Secondary outcome 1: Improved locomotion capacity, increased stability during gait, reduction in gait deviations; evaluated using TUG test and BBS.

Secondary outcome 2: Increased spatial orientation capacity; evaluated by PPA, WHOQOL-Bref, ABC scale and MMSE.

Secondary outcome 3: Reduction in falls;evaluated by FES-I, ABC scale, VADL scale and dynamometer.

Secondary outcome 4: Improved capacity to perform activities of daily living, generating self-confidence that will lead to positive gains in aspects of family, social and professional life; evaluated by WHOQOL-Bref, VAS and VADL scale.

**Possible effects of treatment**

The main clinical manifestation of HSP is a pyramidal pattern of slowly progressive weakness^5^associated with lower limb weakness and progressing to other manifestations, such as dementia, peripheral neuropathy, Parkinson's disease and ataxia.

The aim of VR with virtual reality is to delay the progression of the disease, providing a differentiated, accessible, low-cost treatment option. The use of virtual reality with Nintendo Wii® is a novel, playful, multisensory tool for balance training that engages the patient with enthusiasm and motivation during the continuous sessions. This technological resource is an additional tool to other conventional methods of VR.

**Data analysis**

Data analysis will involve repeated-measures ANOVA with the level of significance set at 5%.

**Control of bias**

The study includes several important methodological resources that will minimize the risk of bias, such as the randomization process, allocation concealment, blinded assessment of the results and intention-to-treat analysis.

**Timeline**

Recruitment of the participants began in April 2019. All participants have been recruited and treatment is expected to be completed by July 2021. The data analysis will be conducted in 2021 and the manuscript will be completed by December 2022.

**Conclusion**

The study will provide a definitive assessment of the effectiveness and cost-benefit of a VR program involving virtual reality to reduce the progressive effect of the disease, providing a differentiated, accessible, low-cost treatment option. The use of virtual reality with Nintendo Wii® is an innovative, playful, multisensory tool for balance training that engages the patient with enthusiasm and motivation during the continuous sessions. This technological resource is an additional tool to other conventional methods of VR.

**Acknowledgments**

Thanks to the service of neurology at the hospital of the federal university of parana for giving permission for the accomplishment of the research and the Coordenação de Aperfeiçoamento de Pessoal de Nível Superior-Brasil (CAPES).

| **Reference** |
| --- |
| 1. Zeigelboim BS, Teive HAG, Sampaio R, Jurkiewicz AL, Liberalesso, PBN Electronystagmography findings in spinocerebellar ataxia type 3 (SCA3) and type 2 (SCA2). *Arq. Neuro-Psiquiatr*. 2011;69(5):760-765, <https://dx.doi.org/10.1590/S0004-282X2011000600007>. 2. Zanoni A, Ganança FF. Realidade virtual nas síndromes vestibulares. [*Rev. Bras Med*](http://portal.revistas.bvs.br/transf.php?xsl=xsl/titles.xsl&xml=http://catserver.bireme.br/cgi-bin/wxis1660.exe/?IsisScript=../cgi-bin/catrevistas/catrevistas.xis\|database_name=TITLES\|list_type=title\|cat_name=ALL\|from=1\|count=50&lang=pt&comefrom=home&home=false&task=show_magazines&request_made_adv_search=false&lang=pt&show_adv_search=false&help_file=/help_pt.htm&connector=ET&search_exp=Rev%20Bras%20Med). 2010; 67(supl.1): 113-116. 3. Zeigelboim BS, Ganança, CF, Ganança, FF. Reabilitação vestibular. In: Zeigelboim BS, Jurkiewicz AL (Org). A Multidisciplinaridade na otoneurologia. 1ed. São Paulo: Roca; 2013a.p.429-44. 4. Zeigelboim BS, Souza SD, Mengelberg H, Teive HAG, Liberalesso PBN. Reabilitação vestibular com realidade virtual na ataxia espinocerebelar.  *Audiol Commun Res.* 2013;18(2),143-147, <https://dx.doi.org/10.1590/S2317-64312013000200013>. 5. Finsterer J, Loscher W, Quasthoff S, Wanschitz J, Aur-Grumbach M, Stevanin G. Hereditary spastic paraplegias with autosomal dominant, recessive, X-linked or maternal trait of inheritance. *J Neurol Sci.* 2012;318:1-18,<https://doi.org/10.1016/j.jns.2012.03.025>. 6. Klimpe S, Schüle R, Kassubek J, Otto S, Kohl Z, Klebe S, Ratzka S, Karle K, Schöls L. Disease severity affects quality of life of hereditary spastic paraplegia patients. *Eur J Neurol*. 2012;19(1):168-171, <https://doi.org/10.1111/j.1468-1331.2011.03443.x>. 7. Fink JK. Hereditary spastic paraplegia: clinico-pathologic features and emerging molecular mechanisms. *Acta Neuropathol*. 2013;126(3):307-328, http://dx.doi.org/ 10.1007/s00401-013-1115-8. 8. Faber I, Servelhere KR, Martinez ARM, D`Abreu A, Lopes-Cendes I, França Jr, MC. Clinical features and management of hereditary spastic paraplegia. *Arq. Neuro-Psiquiatr.* 2014*;* 72(3):219-226, <https://dx.doi.org/10.1590/0004-282X20130248>. 9. Matos VSB, Gomes FS, Sasaki AC. Aplicabilidade da Reabilitação Vestibular nas Disfunções Vestibulares Agudas. *Rev. Equilib. Corporal Saúde*. 2010; 2(1):76-83. 10. Rodrigues TP, Ganança CF, Garcia AP, Caovilla HH, Ganança MM, Ganança FF. Reabilitação vestibular com realidade virtual em pacientes com Doença de Ménière. *Rev. Equilíbr Corporal Saúde*. 2009; 1(1):9-20. 11. [Bruin ED](https://www.ncbi.nlm.nih.gov/pubmed/?term=de%20Bruin%20ED%5BAuthor%5D&cauthor=true&cauthor_uid=20814798), [Schoene D](https://www.ncbi.nlm.nih.gov/pubmed/?term=Schoene%20D%5BAuthor%5D&cauthor=true&cauthor_uid=20814798), [Pichierri G](https://www.ncbi.nlm.nih.gov/pubmed/?term=Pichierri%20G%5BAuthor%5D&cauthor=true&cauthor_uid=20814798), [Smith ST](https://www.ncbi.nlm.nih.gov/pubmed/?term=Smith%20ST%5BAuthor%5D&cauthor=true&cauthor_uid=20814798). Use of virtual reality technique for the training of motor control in the elderly: Some theoretical considerations. Z *Gerontol Geriatric.* 2010;43(4):229–234, <https://doi.org/10.1007/s00391-010-0124-7>. 12. Lord SR; Menz HB, Tiedemann AA. Physiological Profile Approach to Falls Risk Assessment and Prevention. *PhysTher.* 2003; 83(3):237-252, <https://doi.org/10.1093/ptj/83.3.237>. 13. [Li RC](https://www.ncbi.nlm.nih.gov/pubmed/?term=Li%20RC%5BAuthor%5D&cauthor=true&cauthor_uid=16500178), [Jasiewicz JM](https://www.ncbi.nlm.nih.gov/pubmed/?term=Jasiewicz%20JM%5BAuthor%5D&cauthor=true&cauthor_uid=16500178), [Middleton J](https://www.ncbi.nlm.nih.gov/pubmed/?term=Middleton%20J%5BAuthor%5D&cauthor=true&cauthor_uid=16500178), [Condie P](https://www.ncbi.nlm.nih.gov/pubmed/?term=Condie%20P%5BAuthor%5D&cauthor=true&cauthor_uid=16500178), [Barriskill A](https://www.ncbi.nlm.nih.gov/pubmed/?term=Barriskill%20A%5BAuthor%5D&cauthor=true&cauthor_uid=16500178), [Hebnes H](https://www.ncbi.nlm.nih.gov/pubmed/?term=Hebnes%20H%5BAuthor%5D&cauthor=true&cauthor_uid=16500178), [Purcell B](https://www.ncbi.nlm.nih.gov/pubmed/?term=Purcell%20B%5BAuthor%5D&cauthor=true&cauthor_uid=16500178). The development, validity, and reliability of a manual muscle testing device with integrated limb position sensors. *Arch Phys Med Rehabil.*2006;87:411-417, <https://doi.org/10.1016/j.apmr.2005.11.011>. 14. [Kim HM](https://www.ncbi.nlm.nih.gov/pubmed/?term=Kim%20HM%5BAuthor%5D&cauthor=true&cauthor_uid=19181972), [Teefey SA](https://www.ncbi.nlm.nih.gov/pubmed/?term=Teefey%20SA%5BAuthor%5D&cauthor=true&cauthor_uid=19181972), [Zelig A](https://www.ncbi.nlm.nih.gov/pubmed/?term=Zelig%20A%5BAuthor%5D&cauthor=true&cauthor_uid=19181972), [Galatz LM](https://www.ncbi.nlm.nih.gov/pubmed/?term=Galatz%20LM%5BAuthor%5D&cauthor=true&cauthor_uid=19181972), [Keener JD](https://www.ncbi.nlm.nih.gov/pubmed/?term=Keener%20JD%5BAuthor%5D&cauthor=true&cauthor_uid=19181972), [Yamaguchi K](https://www.ncbi.nlm.nih.gov/pubmed/?term=Yamaguchi%20K%5BAuthor%5D&cauthor=true&cauthor_uid=19181972).Shoulder strength in asymptomatic individuals with intact compared with torn rotator cuffs. *J Bone Joint Surg Am*. 2009; 91(2):289-296, <https://doi.org/10.2106/JBJS.H.00219>. 15. Sisto SA, Dyson-Hudson T. Dynamometry testing in spinal cord injury. *J Rehabil Res Dev*. 2007; 44(1):123-136, <https://doi.org/10.1682/jrrd.2005.11.0172>. 16. THE WHOQOL GROUP. Development of the World Health Organization WHOQOL-bref. Quality of Life Assesment. *PsycholMed. 1998*;28:551-8, <https://doi.org/10.1017/s0033291798006667>.      1. Kluthcovsky AC, Kluthcovsky FA. O WHOQOL-bref, um instrumento para avaliar qualidade de vida: uma revisão sistemática. *Rev. Psiquiatr. Rio Gd. Sul,* 2009; 31(3 Suppl.), <https://dx.doi.org/10.1590/S0101-81082009000400007>. 2. Miyamoto ST, Lombardi Junior I, Berg KO, Ramos LR, Natour J. Brazilian version of the Berg balance scale. *Braz. J Med Biol Res*. 2004; 37(9):1411-1421, <https://dx.doi.org/10.1590/S0100-879X2004000900017>. 3. [Marques AP, Mendes YC, Taddei U, Pereira CAB, Assumpção A. Brazilian-portuguese translation and cross cultural adaptation of the Activities-specific Balance Confidence (abc) scale. *Braz J Phys Ther.* 2013;17(2):170-8, PMid:23778771. http://dx.doi.org/10.1590/S1413-35552012005000072.](http://dx.doi.org/10.1590/S1413-35552012005000072) 4. Parry SW, Steen N, Galloway SR, Kenny RA, Bond J. Falls and Confidence Related Quality of Life Outcome Measures in an Older British Cohort. *Postgrad Med J*. 2001;77(904):103-108, http://dx.doi.org/[10.1136/pmj.77.904.103](https://doi.org/10.1136/pmj.77.904.103). 5. Powell LE, Myers AM. The Activities-specific Balance Confidence (ABC) Scale. *J Gerontol Med Sci.*1995; 50A(1):M28-34, https://dx.doi.org/[10.1093/gerona/50a.1.m28](https://doi.org/10.1093/gerona/50a.1.m28). 6. [Lajoie Y, Gallagher SP. Predicting falls within the enderly community: comparison of postural sway, reaction time, the Berg balance scale and the Activities-specific Balance Confidence (ABC) scale for comparing fallers and non-fallers. *Arch Gerontol Geriat*. 2004;38(1):11-26,](file://D:\Users\85592498\Downloads\LAJOIE%20Y,%20GALLAGHER%20SP.%20Predicting%20falls%20within%20the%20enderly%20community:%20comparison%20of%20postural%20sway,%20reaction%20time,%20the%20Berg%20balance%20scale%20and%20the%20Activities-specific%20Balance%20Confidence%20(ABC)%20scale%20for%20comparing%20fallers%20and%20non-fallers.%20Arch%20Gerontol%20Geriatr%20%5binternet%5d.%202004;38(1):11-26%20Available%20from:%20http:\www.ncbi.nlm.nih.gov\pubmed\14599700.) https://dx.doi.org/[10.1016/s0167-4943(03)00082-7](https://doi.org/10.1016/s0167-4943(03)00082-7). 7. Cohen HS, Kimball KT. Development of the vestibular disorders activities of daily living scale. *Arch Otolaryngol Head Neck Surg*. 2000;126(7):881-8, http://dx.doi.org/[10.1001/archotol.126.7.881](https://doi.org/10.1001/archotol.126.7.881). 8. Cohen HS, Kimball KT, Adams AS. Application of the vestibular disor­ders activities of daily living scale. *Laryngoscope*. 2000;110(7):1204-9,<https://doi.org/10.1097/00005537-200007000-00026>. 9. Beaton DE, Bombardier C, Guillemin F, Ferraz MB. Guidelines for the process of cross-cultural adaptation of self-report measures. *Spine (Phila Pa 1976).* 2000;25(24):3186-91, https://doi.org/[10.1097/00007632-200012150-00014](https://doi.org/10.1097/00007632-200012150-00014). 10. [Aratani, MC, Ricci NA, Caovilla HH, Ganança NN. Versão brasileira da Vestibular Disorders Activities of Daily Living Scale (VADL). *Braz J. Otorhinolaryngol*. 2013;79(2):203-11,  http://dx.doi.org/10.5935/1808-8694.20130036](http://dx.doi.org/10.5935/1808-8694.20130036). 11. Folstein, MF, Folstein, SE, Mchugh, PR. Mini Mental State. A practical method for rading the cognitive state of patients for the clinician. *J Psychiatric Res.* 1975;12(3):189-198, http://dx.doi.org/ 10.1016/0022-3956(75)90026-6. 12. Bertolucci, PHF, Brucki, SMD, Campacci, SR, Juliano, Y. O Mini exame do Estado Mental em uma população geral. Impacto da escolaridade. *Arq. Neuro-psiquiatr.* 1994; 52(1):1-7, http://dx.doi.org/10.1590/S0004-282X1994000100001. 13. Brucki SM, Nitrini R, Caramelli P, Bertolucci PH, Ivan H. Okamoto IH. Sugestões para o Uso do Mini-Exame do Estado Mental no Brasil. *Arq. Neuro-psiquiatr.* 2003; 61(3-B):777-81, <http://dx.doi.org/10.1590/S0004-282X2003000500014>. 14. Mitolo M, Salmon DP, Gardini S, Galasko D, Grossi E, Caffarra, P. The new Qualitative Scoring MMSE Pentagon Test (QSPT) as a valid screening tool between autopsy-confirmed dementia with Lewy bodies and Alzheimer’s disease. *J Alzheimers Dis*. 2014; 39(4): 823-832, http://dx.doi.org/ 10.3233/JAD-131403. 15. Thal LJ, Grundman M, Golden R. A correlational analysis of the Blessed Information-Memory-Concentration Test and the Mini-Mental State Exam. *Neurology.* 1986; 36:262-264, https://doi.org/10.1212/WNL.36.2.262. 16. [Paula FL, Alves Junior ED, Prata H. Teste Timed “UpAnd Go”: uma comparação entre valores obtidos em ambiente fechado e aberto. *Fisioter. mov*. 2007;20(4):143-8.](http://www2.pucpr.br/reol/pb/index.php/rfm?dd1=1786&dd99=view&dd98=pb)  <https://periodicos.pucpr.br/index.php/fisio/article/view/18977/18351> 17. Guimarães, LHCT,Galdino DCA, Martins, FLM, et al. Comparação da propensão de quedas entre idosos que praticam atividade física e idosos sedentários. *Rev. Neurocienc*. 2004;12(2):68-72. 18. Yardley L, Beyer N, Hauer K, Kempen G. Piot-Ziegler C, Todd C. Development and initial validation of the Falls Efficacy Scale-International (FES-I). *Age Ageing*. 2005;34(6):614-9, https://doi.org/[10.1093/ageing/afi196](https://doi.org/10.1093/ageing/afi196). 19. Camargos FFO, Dias RC, Dias JMD, Freire MTF. Adaptação Transcultural e Avaliação das propriedades psicométricas da Falls EfficacyScale – Internacional em idosos Brasileiros (FES-I-BRASIL). *Braz* *J Phys Ther*. 2010; 14(3):237-43, http://dx.doi.org/10.1590/S1413-35552010000300010. 20. Carvalho DS, kowacs PA. Avaliação da intensidade de dor. *Migrâneas cefaléias*. 2006; 9(4): 164-68. |
