# Supplementary material for: Balance rehabilitation with a virtual reality protocol for patients with hereditary spastic paraplegia: Protocol for a clinical trial
Source: PLoS One. 2021 Apr 1;16(4):e0249095. doi: 10.1371/journal.pone.0249095 (PMC8016341; doi:10.1371/journal.pone.0249095)
Supplement: Attachment — Submitted filename: Response to Reviewers.docx [file pone.0249095.s004.docx]

Dear Sir,

Thanks for the reviewer's comments about our article. All corrections were made and the questions answered.

**REVISION NOTES**

**REVIEWER 1**

Abstract:
1. Start with the subtitle – background All suggestions have been considered and changed in the text.

2. Mention clearly the duration of outcome measurement. All suggestions have been considered and changed in the text. (line 385-387).

3. Mention the reports with 95% CI with p values. All suggestions have been considered and changed in the text.

4. Avoid abbreviations in the conclusion. All suggestions have been considered and changed in the text.

Introduction
1. The introduction part is too short and didn’t mention about important key points. All suggestions have been considered and changed in the text.
2. How come your study is differed from reference 9 and 10? The present study deals with hereditary spastic paraplegia, a degenerative disease involving the central nervous system and this differs from the references cited [9 and 10] in that different diseases are evaluated and different equipment is used in virtual reality rehabilitation. In the references cited, chronic diseases and Ménière's disease, determined by tinnitus, vertigo and hearing loss, were evaluated in paroxysmal episodes without involvement of the central nervous system, with endolymphatic hydrops being considered as the pathophysiological basis of this disease. The equipment used was the Balance Rehabilitation Unit (BRUTM).

3. The research question is not formulated with suitable references.

The objective of this study as well as the question is to determine the benefits of VR involving virtual reality, comparing the results of pre- and post-intervention evaluations in individuals with hereditary spastic paraplegia, as the great motivation for carrying out this study was based on the fact there are no studies like the one mentioned in the introduction. "No randomized or nonrandomized studies related to VR with virtual reality in patients with HSP have been published in the PubMed electronic databases, including Medline, Scielo, Embase, Scopus and Web of Science".
4. Add more recent researches related virtual reality and its effects. All suggestions have been considered and changed in the text.

5. Define the clinical significance of this review in related to researchers, clinicians and patients. This research is a clinical trial not a review.

Methods
6. Mention clearly who is diagnosing and selecting the patients for the study. All suggestions have been considered and changed in the text.
7. Make the inclusion and exclusion criteria in a paragraph format. All suggestions have been considered and changed in the text.
8. Missing of references for intervention procedures. References 2, 4, 10, 11, 12, 13 and 14 include intervention procedures with virtual reality.
9. The reference for otolaryngolic evaluation and vestibular assessment. All suggestions have been considered and changed in the text.
10. The selection criteria should be more specific – (inclusion and exclusion) All suggestions have been considered and changed in the text.
11. Mention the method and referral study used for calculating the sample size. All suggestions have been considered and changed in the text.
(line 373).

**REVIEWER 2**

Minor revisions:

1. ABSTRACT: The abstract does not clearly indicate that the proposed study will be a randomized, controlled clinical trial. The methods section is composed with awkward language. For instance, the methods section contains verbs that are past tense while others are present tense. All suggestions have been considered and changed in the text.

2. Provide a more comprehensive statistical analysis plan. State the statistical methods that will be used to summarize the outcomes. Indicate the types of plots that will illustrate the results. Indicate that group I will be compared to group II using an ANOVA. Consider including an alternative analysis to ANOVA since the somewhat restrictive requirements and assumption of ANOVA may not be met. As an alternative consider a mixed linear model.

All suggestions have been considered and changed in the text.
