# Supplementary material for: Balance rehabilitation with a virtual reality protocol for patients with hereditary spastic paraplegia: Protocol for a clinical trial
Source: PLoS One. 2021 Apr 1;16(4):e0249095. doi: 10.1371/journal.pone.0249095 (PMC8016341; doi:10.1371/journal.pone.0249095)
Supplement: Attachment — Submitted filename: Response to Reviewers 2.docx [file pone.0249095.s005.docx]

March, 1^st^.

Dear Sir,

Thanks for the reviewer's comments about our article. All corrections were made and the questions answered.

**REVISION NOTES**

**REVIEWER 1**

1. Results part should be more informative including CI 95% with p values. Thank you for your comment, but we need to inform you that the results will be informed after the execution of the protocol of this clinical trial.
2. Present the article in a simple past tense than future tense. The text in general is in the future because it is a clinical trial protocol.
3. Include latest references in the field of virtual reality training. All suggestions have been considered and made the reference insertion number 10.
4. Please provide the definitive conclusion. In the same way that we do not have the results definitively, we also do not provide a definitive conclusion. Because this is a trial protocol.

**REVIEWER 2**

I have no additional comments. Thank you for your comments.
